# Supplementary material for: Functionalized MXene ink enables environmentally stable printed electronics
Source: Nat Commun. 2024 Apr 24;15:3459. doi: 10.1038/s41467-024-47700-y (PMC11043420; doi:10.1038/s41467-024-47700-y)
Supplement: Supplementary file 1 — Supplementary Information [file 41467_2024_47700_MOESM1_ESM.pdf]

Supplementary Information

Functionalized MXene Ink Enables Environmentally Stable Printed Electronics

Tae Yun Ko[1,2,3,¶], Heqing Ye[4,5,¶], G. Murali[6,7,¶], Seul-Yi Lee[8,¶], Young Ho Park[6,7], Jihoon Lee[6,7], Juyun Lee[1,2,9], Dong-Jin Yun[10], Yury Gogotsi[11], Seon Joon Kim[1,2,12,*], Se Hyun Kim[5*], Yong Jin Jeong[7,13,*], Soo-Jin Park[8,*], Insik In[6,7,*]

[1]Materials Architecturing Research Center, Korea Institute of Science and Technology, 5, Hwarang-ro 14-gil, Seongbuk-gu, Seoul 02792, South Korea

[2]Convergence Research Center for Solutions to Electromagnetic Interference in Future-mobility, Korea Institute of Science and Technology, 5, Hwarang-ro 14-gil, Seongbuk-gu, Seoul 02792, South Korea

[3]Nanoplexus Solutions Ltd, Graphene Engineering Innovation Centre, Masdar Building, Sackville Street, Manchester M1 3BB, UK

[4]School of Flexible Electronics (SoFE) and Henan Institute of Flexible Electronics (HIFE), Henan University, 379 Mingli Road, Zhengzhou 450046, China

[5]School of Chemical Engineering, Konkuk University, Seoul 05029, South Korea

[6]Department of Polymer Science and Engineering, Chemical Industry Institute, Korea National University of Transportation, Chungju 27469, South Korea

[7]Department of IT-Energy Convergence (BK21 FOUR), Korea National University of Transportation, Chungju 27469, South Korea

[8]Department of Chemistry, Inha University, Inharo 100, Incheon 22212, South Korea

[9]Department of Materials Science and Engineering, Korea University, 145, Anam-ro, Seongbuk-gu, Seoul 02841, South Korea

[10]Analytical Science Laboratory of Samsung Advanced Institute of Technology (SAIT), Suwon 16678, South Korea

[11]Department of Materials Science and Engineering and A. J. Drexel Nanomaterials Institute, Drexel University, Philadelphia, Pennsylvania 19104, United States

[12]Division of Nanoscience and Technology, KIST School, University of Science and Technology, 5, Hwarang-ro 14-gil, Seongbuk-gu, Seoul 02792, South Korea

[13]Department of Materials Science and Engineering, Korea National University of Transportation, Chungju 27469, South Korea

[¶]Tae Yun Ko, Heqing Ye, G. Murali, and Seul-Yi Lee are the first authors of this manuscript.

*Correspondence: in1@ut.ac.kr (Insik In), sjpark@inha.ac.kr (Soo-Jin Park), yjjeong@ut.ac.kr (Yong Jin Jeong), shkim97@konkuk.ac.kr (Se Hyun Kim), Seonjkim@kist.re.kr (Seon Joon Kim)

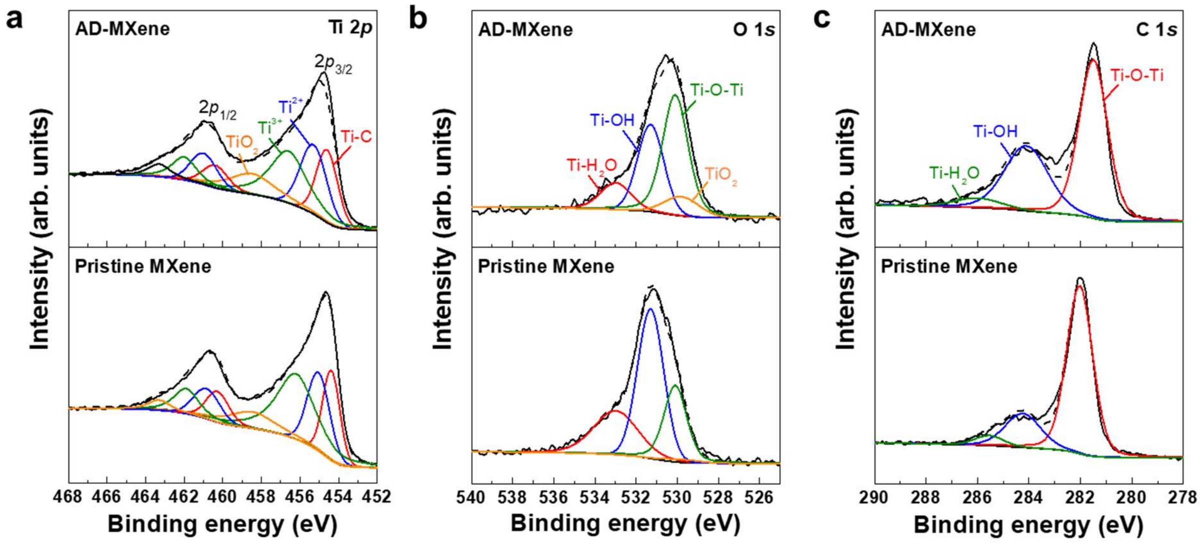

Supplementary Fig. 1. XPS spectra of pristine MXene and AD-MXene. **a-c,** Ti 2*p* (**a**), O 1*s* (**b**), and C 1*s* (**c**).

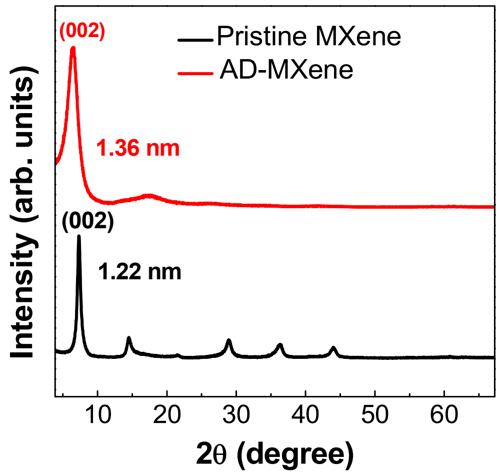

Supplementary Fig. 2. XRD patterns of pristine MXene and AD-MXene.

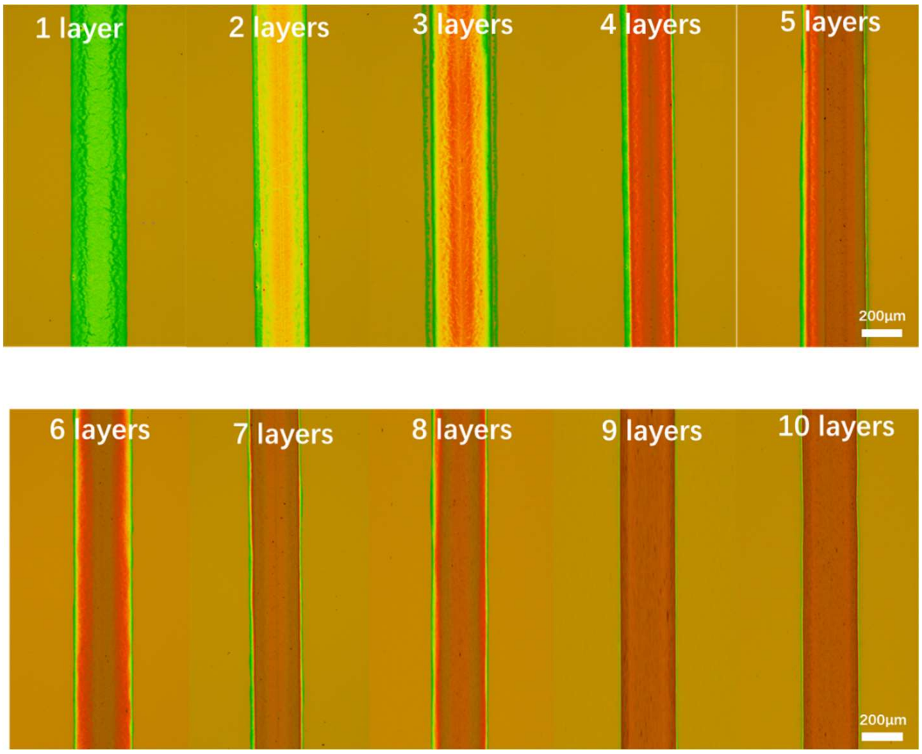

Supplementary Fig. 3. Optical micrographs of AD-MXene lines fabricated with different number of EHD printing cycles.

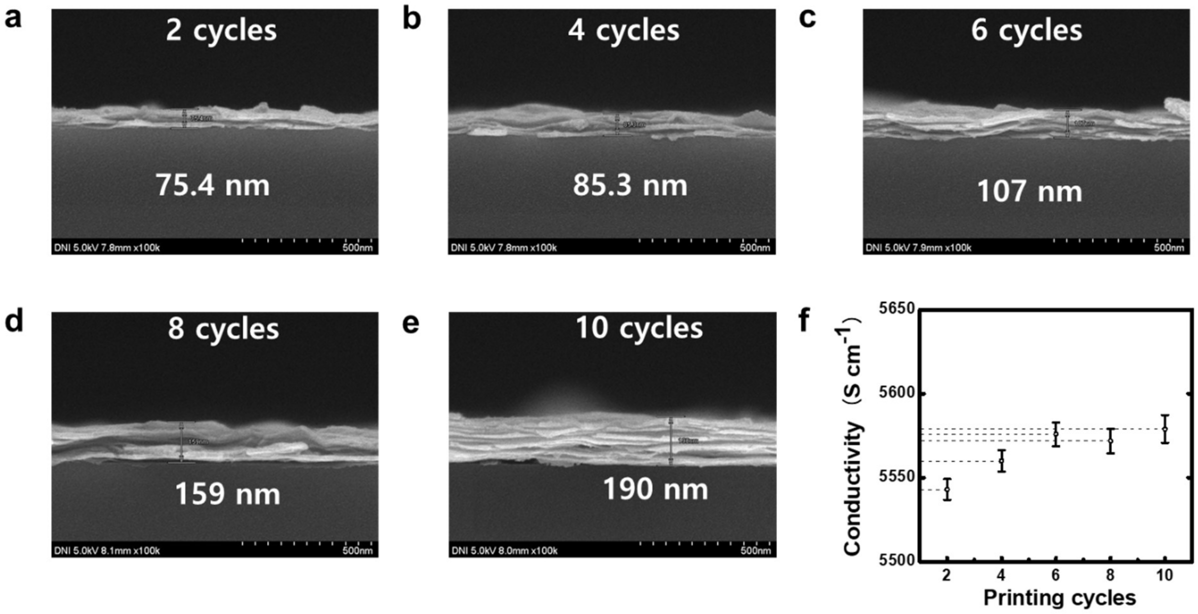

Supplementary Fig. 4. a-e, Cross-sectional FE-SEM images of AD-MXene lines fabricated with different number of printing cycles. **f,** Electrical conductivities of AD-MXene lines fabricated with 1-10 printing cycles. The error bars in this figure represent the standard deviations of three parallel tests.

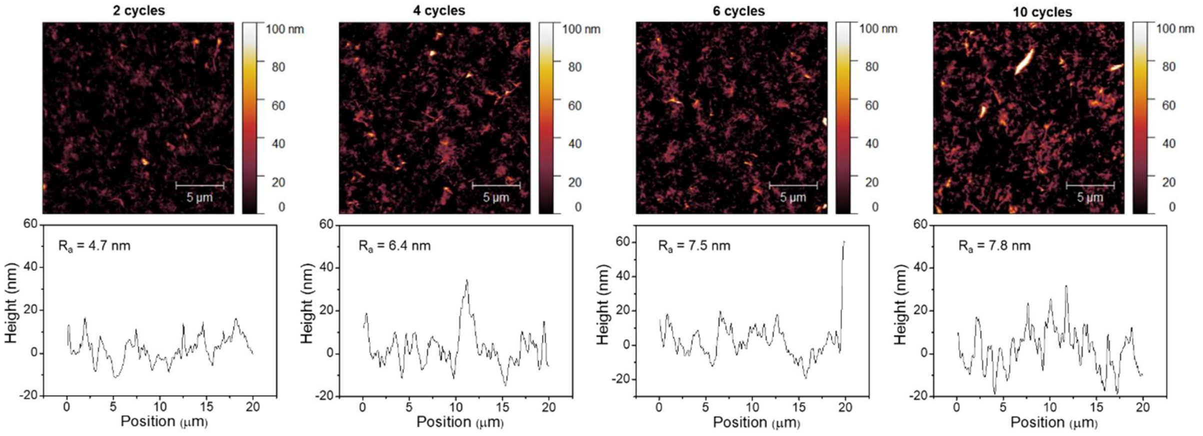

Supplementary Fig. 5. AFM images and surface roughness of AD-MXene lines fabricated with different number of printing cycles.

.

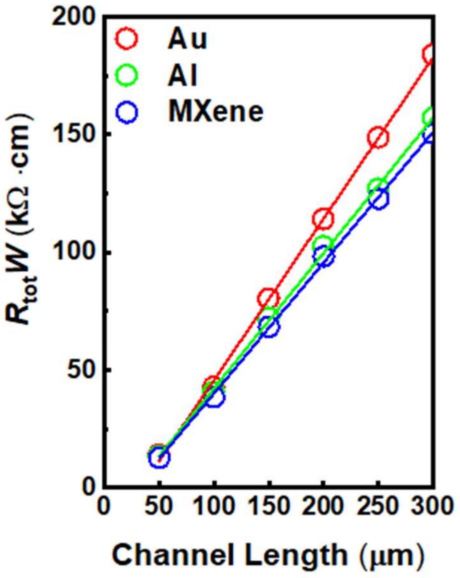

Supplementary Fig. 6. Width-normalized total resistance as a function of channel length for TFTs with Au, Al, and AD-MXene electrodes.

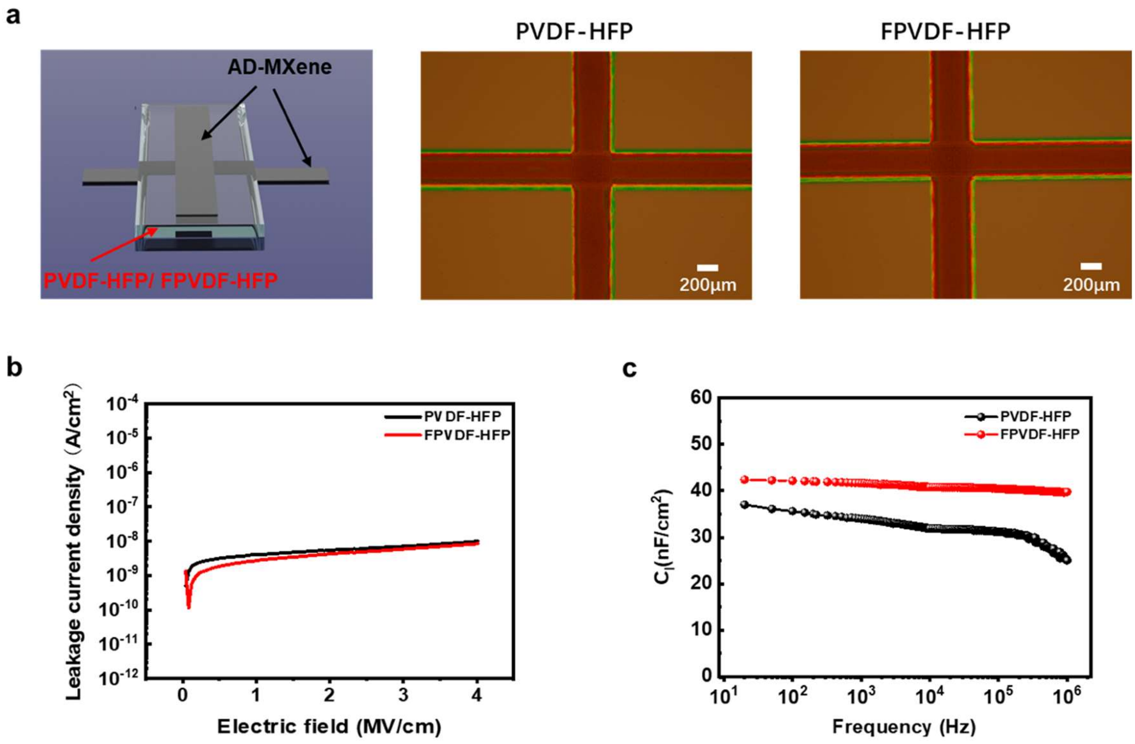

Supplementary Fig. 7. a, Schematic representation (left) and top-view optical images (middle and right) of MIM capacitors with EHD-printed AD-MXene and PVDF-HFP or FPVDF-HFP layers. **b,c,** Leakage current density as a function of applied electric field strength (**b**) and areal capacitance (Ci) as a function of frequency (**c**) plots of MIM capacitors with PVDF-HFP and FPVDF-HFP dielectrics.

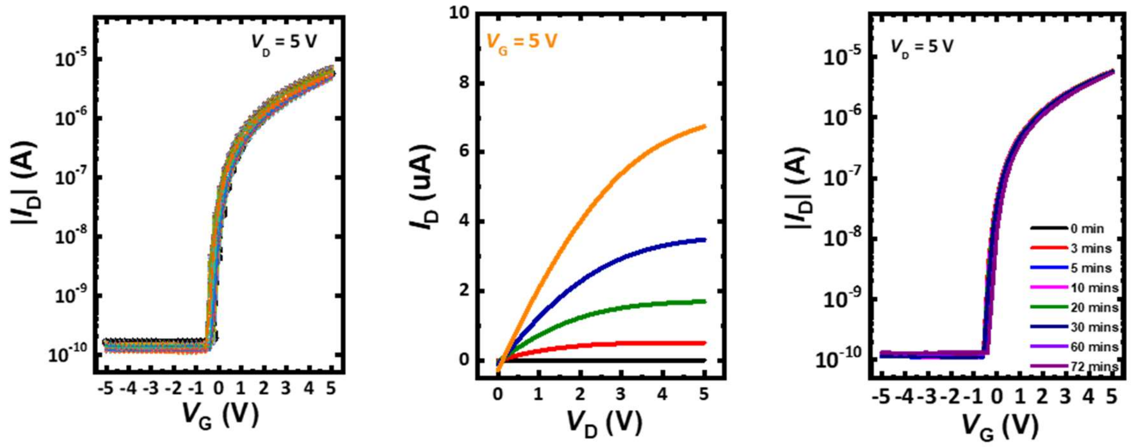

Supplementary Fig. 8. Transfer curves of 64 TFT devices; output characteristics and gate bias stress stabilities of an individual TFT in the array.

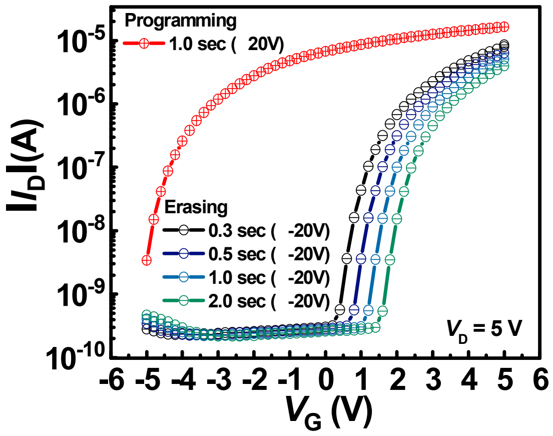

Supplementary Fig. 9. Transfer curves after applying programming level (20 V) for 1 s; and erasing level (-20 V) for various time durations (0.3, 0.5, 1.0, and 2.0 s).
